# Comparison of Data Normalization Strategies for Array-Based MicroRNA Profiling Experiments and Identification and Validation of Circulating MicroRNAs as Endogenous Controls in Hypertension

**DOI:** 10.3389/fgene.2022.836636

**Published:** 2022-03-31

**Authors:** Lakshmi Manasa S. Chekka, Taimour Langaee, Julie A. Johnson

**Affiliations:** ^1^ Department of Pharmacotherapy and Translational Research and Center for Pharmacogenomics and Precision Medicine, University of Florida, Gainesville, FL, United States; ^2^ Division of Cardiovascular Medicine, Department of Medicine, University of Florida, Gainesville, FL, United States

**Keywords:** hypertension, endogenous control, data normalization, plasma microRNA, circulating microRNA

## Abstract

**Introduction:** MicroRNAs are small noncoding RNAs with potential regulatory roles in hypertension and drug response. The presence of many of these RNAs in biofluids has spurred investigation into their role as possible biomarkers for use in precision approaches to healthcare. One of the major challenges in clinical translation of circulating miRNA biomarkers is the limited replication across studies due to lack of standards for data normalization techniques for array-based approaches and a lack of consensus on an endogenous control normalizer for qPCR-based candidate miRNA profiling studies.

**Methods:** We conducted genome-wide profiling of 754 miRNAs in baseline plasma of 36 European American individuals with uncomplicated hypertension selected from the PEAR clinical trial, who had been untreated for hypertension for at least one month prior to sample collection. After appropriate quality control with amplification score and missingness filters, we tested different normalization strategies such as normalization with global mean of imputed and unimputed data, mean of restricted set of miRNAs, quantile normalization, and endogenous control miRNA normalization to identify the method that best reduces the technical/experimental variability in the data. We identified best endogenous control candidates with expression pattern closest to the mean miRNA expression in the sample, as well as by assessing their stability using a combination of NormFinder, geNorm, Best Keeper and Delta Ct algorithms under the Reffinder software. The suitability of the four best endogenous controls was validated in 50 hypertensive African Americans from the same trial with reverse-transcription–qPCR and by evaluating their stability ranking in that cohort.

**Results:** Among the compared normalization strategies, quantile normalization and global mean normalization performed better than others in terms of reducing the standard deviation of miRNAs across samples in the array-based data. Among the four strongest candidate miRNAs from our selection process (miR-223-3p, 19b, 106a, and 126-5p), miR-223-3p and miR-126-5p were consistently expressed with the best stability ranking in the validation cohort. Furthermore, the combination of miR-223-3p and 126-5p showed better stability ranking when compared to single miRNAs.

**Conclusion:** We identified quantile normalization followed by global mean normalization to be the best methods in reducing the variance in the data. We identified the combination of miR-223-3p and 126-5p as potential endogenous control in studies of hypertension.

## Introduction

Hypertension (HTN) is a major public health burden, affecting more than 1 billion individuals worldwide (World Health Organization, 2021). It is a major yet modifiable risk factor for myocardial infarction, stroke, heart failure, and kidney failure (Kokubo and Iwashima, 2015). Most cases (95%) of HTN are essential or primary or idiopathic HTN, where the underlying cause is unknown (Jusic and Devaux, 2019). Essential HTN is a complex and heterogenous phenotype involving several tissues and pathways and exhibits large interpatient variability in the pathophysiology and response to different therapeutic agents (Nardin et al., 2018; Shih and O'Connor, 2008). Despite extensive efforts to understand the pathogenesis of HTN, the underlying cellular and molecular mechanisms remain largely elusive.

MicroRNAs (miRNAs) are a subclass of noncoding RNAs that act via sequence-specific interaction with messenger RNAs (mRNAs), causing either degradation or translational repression of target mRNA (Bartel, 2004). They are involved in regulation of virtually all cellular functions, and their deregulation is implicated in a variety of diseases like cancer (Ratti et al., 2020) and cardiovascular diseases (Das et al., 2020) including HTN (Shi et al., 2015). MiRNAs from different tissues are often released into the circulatory system with a potential role in cross-tissue communication (Mori et al., 2019). Altered circulating miRNA levels were observed in various disease states and often studied as noninvasive predictive biomarkers for disease prognosis or response to therapy (Condrat et al., 2020). Because circulating miRNAs are often found enclosed in extracellular vesicles or bound to protein complexes, they are resistant to degradation by RNAses (Li and Zhang, 2015) and to extreme changes in temperature and pH, making them promising biomarker candidates. There is accumulating evidence on the role of circulating miRNAs and their potential as prognostic biomarkers in HTN (Romaine et al., 2016; Jusic and Devaux, 2019). The studies from our laboratory demonstrated the association of certain miRNAs with response to antihypertensive therapies, suggesting their potential future utility in precision medicine (Solayman et al., 2019).

An important challenge in clinical translation of circulating miRNA biomarkers is the low reproducibility across studies (De Ronde et al., 2017). MiRNA biomarker identification often starts with a high-throughput screening (e.g., microarray panel containing hundreds of miRNAs), after which the most promising candidates are validated by quantitative polymerase chain reaction (qPCR) measurements in independent samples. Although qPCR is a sensitive method, challenges arise when working with target quantities near the detection limit of qPCR, as is the case for many circulating miRNAs. This leads to missing data, which is handled and interpreted differently among studies, leading to differences in findings (De Ronde et al., 2017). Additionally, experimentally introduced artifacts, e.g., starting sample amount, collection and storage conditions, and miRNA extraction/transcription efficiency, profoundly affect the final results of qPCR, eventually masking the true associations. It is important to normalize the data to reduce this analytical variability to obtain the most reliable and reproducible results (Faraldi et al., 2019).

Currently, a variety of normalization techniques are used for array-based methods, such as mean-centering/global normalization, quantile normalization, normalization with a restricted set of highly expressed miRNAs (restricted mean centering), standard housekeeping miRNA/endogenous control normalization, and exogenous control normalization, but each has its own advantages and disadvantages. In cases where a few candidate miRNAs are being profiled, which is most likely the case in clinical settings, only standard housekeeping miRNA and exogenous control methods can be used to normalize the reverse-transcription–qPCR (RT-qPCR) data.

Exogenous oligonucleotides (such as cel-miR-39, cel-miR-54 or cel-miR-238, and ath-miR159a) (Faraldi et al., 2019) are often used as external controls and added at known concentrations to the biological samples before RNA extraction. While they are useful to correct for the RNA extraction efficiency and reverse transcription efficiencies of the kits used, they cannot account for the other previously described intrinsic variables to which they are not exposed. Endogenous miRNAs might be considered as optimal references/normalizers since their expression is affected by the same variables as the target miRNAs. One of the most commonly used endogenous miRNAs-based normalization strategy in experiments assaying large numbers of miRNAs is global mean normalization that involves the averaged Ct (cycle threshold) value of all the analyzed miRNAs. Quantile normalization can also be used for array-based methods to reduce the technical variabilities in the experiments, but it has the disadvantage of reducing/masking the biological variations of interest (Hicks and Irizarry, 2014). In candidate miRNA profiling, it is essential to identify and use a single or a combination of endogenous controls or housekeeping microRNAs when analyzing a small number of miRNAs, and it is critical to identify a suitable housekeeping miRNA to ensure accurate results.

While several endogenous miRNAs, such as U6 and miR-16, are often used as reference miRNAs for normalizing tissue/cellular miRNA expression data (Das et al., 2016), there is no consensus on a circulating miRNA normalizer. Research has shown that U6 is unstable in plasma. Additionally, since plasma miRNA profiles are oftentimes altered in disease states, it is unlikely to discover a universal circulating miRNA normalizer. Thus, it is important to identify a housekeeping miRNA(s) for a specific disease and oftentimes specific to the experiment. To the best of our knowledge, there is no consensus on endogenous control miRNAs for RT-qPCR analysis of plasma miRNAs in HTN.

In this study, we aim to compare different normalization strategies for array-based data as well as identify and validate suitable endogenous circulating miRNA controls for normalization of candidate miRNA studies in HTN, with the objective of standardizing the analysis and to improve reproducibility across studies.

## Methods

### Study Cohort and Sample Collection

Biological samples and clinical data used in this study were collected as part of the PEAR (Pharmacogenomic Evaluation of Antihypertensive Responses) trial (clinicaltrials.gov #NCT00246519). The design and objectives of the study have been previously described (Hicks and Irizarry, 2014). In brief, PEAR was a multicenter, randomized clinical trial with the primary aim of evaluating the role of genetic variability on blood pressure (BP) response in Hydrochlorothiazide and/or atenolol treated patients. All participants (*n* = 768) were 17–65 years of age and had mild-to-moderate uncomplicated HTN. After an antihypertensive drug washout period of 4–6 weeks, the baseline biological samples were collected in a fasting state.

The study was approved by the institutional review boards at each site, and all participants gave written informed consent.

We selected 36 European Americans (EAs) with uncomplicated HTN from the PEAR study to profile genome-wide plasma miRNAs using a microarray-based method. We compared a variety of data normalization strategies to identify the ones that best reduced the variability in the data. We further identified a list of endogenous control miRNAs with potential utility as housekeeping miRNAs and validated them in a cohort of 50 African Americans (AA) with uncomplicated HTN from the same study.

### Comparison of Normalization Strategies

The following methods of normalization were compared:

Global mean normalization: normalized Ct for each miRNA for each sample is calculated by subtracting the mean of all analyzed miRNAs in that sample from the raw Ct of that miRNA.

Mean centering_unimputed: this is similar to global mean but uses the mean of only the expressed miRNAs, omitting the missing values.

Mean centering restricted or MCR normalization: normalization with a restricted set of miRNAs that are expressed across all samples (zero missingness in data).

Quantile normalization: this method assumes that the statistical distribution of each sample is the same. Normalization is achieved by forcing the observed distributions to be the same as the average distribution, obtained by taking the average of each quantile across samples which is used as the reference.

Endogenous control normalization: normalized Ct is obtained by subtracting the endogenous control miRNA Ct from the raw Ct of the miRNA in each sample.

To compare the above methods, we plotted the standard deviation (SD) of each miRNA across all samples for the raw data and normalized data using each of the above methods, to identify the methods that best reduced variation in the data.

To identify suitable endogenous controls/housekeeping miRNAs, we followed the steps stated below.

### Endogenous Control Selection

Though many strategies have been proposed to select the best endogenous control from miRNA arrays (Vandesompele et al., 2021), recent proposals indicate that the similarity between the values of an endogenous control and the global mean (which is considered the gold standard by many) is one of the best approximations (Santamaria-Martos et al., 2019). Hence, we selected the miRNAs with the lowest variability (those with smallest SD) after normalization with the global mean (Santamaria-Martos et al., 2019). While it is important that the endogenous control closely represents the mean miRNA expression of the sample, it is also important that endogenous control is highly and consistently expressed across all samples to allow accurate quantification and normalization. We fed the raw data after initial quality control into the Reffinder (Xie et al., 2012) software, a tool that evaluates and screens reference genes or miRNAs using the currently available major computational programs NormFinder (Andersen et al., 2004), Delta Ct method (Silver et al., 2006), geNorm (Vandesompele et al., 2002), and BestKeeper (Pfaffl et al., 2004) algorithms to compare and rank the tested candidate reference genes or miRNAs. Based on the rankings from each program, it assigns an appropriate weight to an individual miRNA and calculates the geometric mean of their weights for the overall final ranking.

To dissect out the potential impact of age and gender on the miRNA expression levels, we conducted a sensitivity analysis with residuals of miRNA levels after regressing out age and gender. Firstly, the global mean-normalized miRNA levels were regressed with age and gender, and the resultant residuals were used to calculate the SD to identify miRNAs closest to the global mean and with lowest variability. Secondly, raw miRNA Ct data were regressed with age and gender. The resultant residuals were fed into the Reffinder software to identify the miRNAs with best stability rankings.

The top four miRNAs that closely resembled the mean expression of the sample as well as with the best comprehensive ranking from Reffinder were taken forward for validation by single-tube RT-qPCR–based assays in the African American cohort.

### Endogenous Control Validation

We profiled the four control miRNAs along with nine additional miRNAs that were selected for an unrelated project, using individual RT-qPCR. The raw Ct values after initial quality control were fed into Reffinder to see if each selected endogenous control still showed high comprehensive ranking, confirming it to be a good housekeeping miRNA. In our study, the validation cohort was selected in order to validate the findings in a different ancestry group (African Americans) from the discovery cohort (European Americans), considering that miRNA expression differences exist by ancestry (Dluzen et al., 2016). For the candidate miRNAs that were validated with the highest comprehensive ranking, we further tested using Reffinder, if the combination of miRNAs was a better endogenous control than single miRNAs.

### RNA Extraction and microRNA Profiling

The plasma was separated from the baseline blood samples collected in EDTA vacutainer tubes and stored in aliquots at −80°C for long-term storage. About 100 μl of the plasma samples were used to extract the total RNA by the MagMAX mirVana Total RNA Isolation Kit (Thermo Fisher Scientific, CA) using the manufacturer’s protocol, in 30 μl of elution buffer. After checking the quantity and quality of the extracted total RNA, about 100 ng of the RNA was used to reverse transcribe to cDNA using the TaqMan reverse transcription kit and Megaplex Primer Pools A and B, followed by preamplification using TaqMan PreAmp Master Mix and Megaplex PreAmp Primer Pools A and B (Applied Biosystems, Thermo Fisher Scientific, CA). The pre-amplified product was diluted and added with TaqMan PCR Master Mix onto the TaqMan OpenArray Human MicroRNA Panel for quantification on QuantStudio™ 12K Flex system using real-time qPCR technique. The TaqMan OpenArray Human MicroRNA Panel (Applied Biosystems, Thermo Fisher Scientific, CA) tests for 754 miRNAs.

Raw data obtained from QuantStudio were filtered of those with <1.1 Amp Score and < 0.7 Cq Confidence scores (Cq Conf). Samples that showed very low miRNA expression/detection and microRNAs with missing Cts in ≥50% of samples were excluded from further analysis. Among the remaining samples and miRNAs that were considered for analysis, missing values were replaced with minimum cycle threshold (Ct) of 40.

### Reverse Transcription–Quantitative Polymerase Chain Reaction Validation

The total RNA was extracted from 100 µl of baseline plasma as described above. The samples were normalized to 10 ng/µl total RNA concentration. For further steps, 2 µl of the normalized samples were used. TaqMan™ Advanced miRNA cDNA Synthesis Kit was used to perform poly(A) tailing, adapter ligation, RT reaction, and preamplification using the manufacturer’s protocols for TaqMan Advanced miRNA single-tube assays. After 1:10 dilution of the pre-amplified product, we performed PCR reactions in 10-µl volumes in triplicate using TaqMan™ Fast Advanced Master Mix and the selected TaqMan™ Advanced miRNA Assays.

The Ct values with Amp Score <1.1 were filtered out. This lower cutoff, as compared to discovery, was considered appropriate since qPCR was done in triplicate that would provide sufficient confidence on miRNA expression and Ct values. The average of the triplicate Cts was used for analysis. Samples with low miRNA expression/detection and miRNAs with missing Cts in >50% of the samples were excluded from the analysis. Missing Cts were replaced with 40.

### Statistical Analysis

R studio (version 3.6.1) and SPSS were used for statistical analysis.

## Results

### Patient Characteristics

The clinical and demographic characteristics of the patients are shown in Table 1. Patients were middle-aged (<60 years), overweight–obese. After filtering miRNAs for Amp Score and Cq Confidence threshold, we excluded six samples with overall low miRNA expression/detection. Of the 754 miRNAs tested, 346 unique miRNAs were detected in the plasma in at least one of the samples. An average of 108 miRNAs were detected in each sample. Only 81 miRNAs were detected consistently across samples with <50% missingness. Further analysis was conducted on these 81 miRNAs from 30 samples.

**TABLE 1:** Demographics.

Characteristics	Discovery (*n* = 36)	Validation (*n* = 50)
Age (years)	47.8 ± 9.9	48.8 ± 6.3
Females (n,%)	16 (44%)	29 (58%)
BMI (kg/m^2^)	30.0 ± 5.1	31.9 + 5.6
Baseline DBP (mmHg)	94.2 ± 4.6	95.4 + 4
Baseline SBP (mmHg)	145.3 ± 10	143.2 + 11
Hip circumference (cm)	109.2 ± 10.8	111.8 + 11.3
Waist circumference (cm)	100.9 ± 16.5	104.9 + 14.7

Note: Data are normally distributed when tested by Shapiro–Wilk test. Hence, mean and standard deviation were used to describe the data distribution.

DBP, Diastolic Blood Pressure; SBP, Systolic Blood Pressure; BMI, Body Mass Index.

### Comparison of Normalization Strategies

**FIGURE 1:**
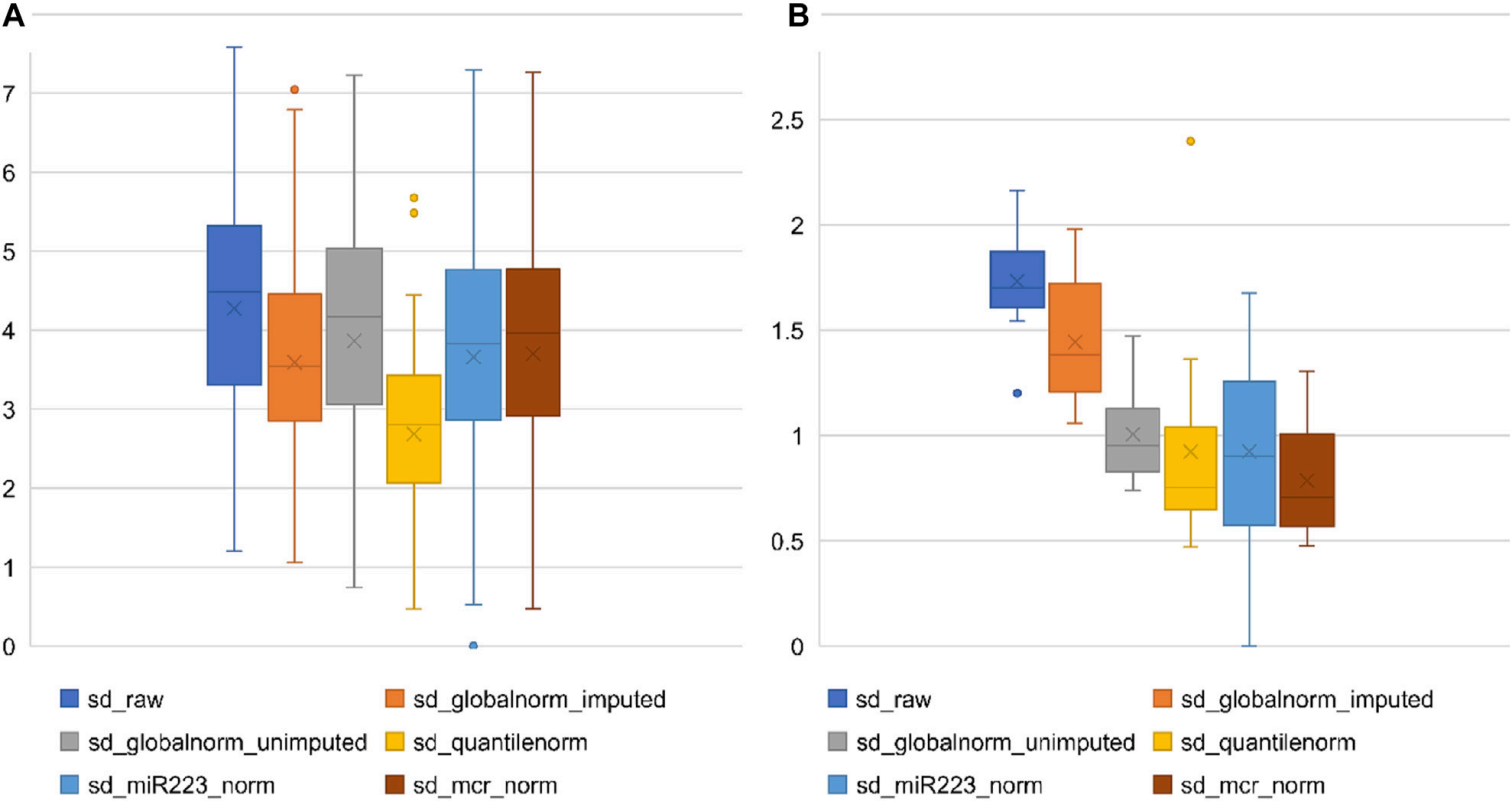
Effect of normalization methods on variation of miRNA expression in microarray data. **(A)** Each box represents the distribution of standard deviation of all analyzed miRNAs (*n* = 81) or **(B)** restricted set of miRNAs (Restricted miRNAs; *n* = 13) on the TaqMan array, calculated separately across all samples. *Y*-axis represents standard deviation (sd).

Figures 1A,B present the effect of different normalization strategies on the expression of all miRNAs and on a restricted set of 13 miRNAs, respectively, that were consistently detected across all samples. For the purpose of this comparison, to represent endogenous control normalization, we used miR-223-3p that showed expressions closest to the global mean (Table 2). We noticed that all the normalization strategies reduced the variation in the data to some extent (seen as reduction in the mean SD of miRNAs from the raw SD). Quantile normalization followed by global mean normalization (global mean after imputation of missing values) showed the greatest reduction in variation. Normalization with the single miR-223 performed similar to normalization with the mean of the restricted set of 13 consistently expressed miRNAs (MCR_norm). The effect of different types of normalization on the well-expressed miRNAs was slightly different from the effect on all miRNAs (as seen in Figure 1A vs. Figure 1B). The MCR method performed best, followed by quantile normalization. Supplementary Figure S1 shows the trend of different normalizers across patient samples.

**TABLE 2:** Endogenous control candidate miRNAs with expression closest to the global mean.

Target	SD
hsa-miR-223_002295_A	1.06
hsa-miR-19b_000396_A	1.13
hsa-miR-126#_000451_B	1.21
hsa-miR-106a_002169_A	1.21
hsa-miR-24_000402_A	1.22
hsa-miR-17_002308_A	1.24
hsa-miR-484_001821_A	1.38
hsa-miR-191_002299_A	1.42
hsa-miR-30c_000419_A	1.51
hsa-miR-320_002277_A	1.59
hsa-miR-150_000473_A	1.86
hsa-miR-331_000545_A	1.95
hsa-miR-146a_000468_A	1.96
U6 rRNA_001973_B	1.98

SD, standard deviation. MicroRNAs with the values most similar to the global mean expression were determined by normalizing the data set with the global mean to select the miRNAs with the smallest standard deviation.

### Candidate Endogenous Controls

Table 2 shows the ordered list of miRNAs which closely resemble the global mean, i.e., with the smallest SD across samples after global mean normalization. We present a list of those with mean SD <2 ∆Ct (Table 2). hsa-miR-223-3p, hsa-miR-19b, hsa-miR-126-5p, and hsa-miR-106a were the top four miRNAs that showed the least SD after normalization with the global mean.

Table 3 shows the rankings for the most stably expressed miRNAs according to different software programs, along with the Reffinder comprehensive ranking. The same four miRNAs: 19b, 223-3p, 106a, and 126-5p showed the highest stability (represented by the smallest stability ranking).

**TABLE 3:** Endogenous control candidates stability ranking in microarray data

Method/Rank	1	2	3	4	5	6	7	8	9	10	11
Delta CT	miR-223	miR-19b	miR-106a	miR-17	miR-24	miR-126-5p	miR-484	miR-191	miR-30c	miR-320	miR-150
BestKeeper	miR-150	miR-126-5p	U6_rR40	miR-320	miR-106a	miR-19b	miR-484	miR-17	miR-24	miR-223	miR-191
NormFinder	miR-223	miR-19b	miR-126-5p	miR-106a	miR-24	miR-17	miR-484	miR-191	miR-30c	miR-320	miR-150
geNorm	miR-106a| miR-19b		miR-223	miR-17	miR-24	miR-191	miR-484	miR-320	miR-126-5p	miR-30c	miR-150
Comprehensive ranking	**miR-19b**	**miR-223**	**miR-106a**	**miR-126-5p**	miR-17	miR-24	miR-150	miR-484	miR-320	miR-191	U6_rR40

Top four miRNAs: miR-19b, miR-223, miR-106a, and miR-126-5p were moved forward for validation.

We repeated the selection process with residuals after regressing out age and gender from the miRNA expression levels (data not presented). Neither the order of top miRNAs closest to the global mean nor the results from the Reffinder differed from our original results. Thus confirming the consistency of the selected miRNAs as normalizers, as they were not affected by changes in age or gender.

### Validation of Endogenous Controls in Cross Ancestry Cohort

The four miRNAs: 19b, 223-3p, 106a, and 126-5p were tested for stability among a custom set of 13 miRNAs (that were selected for a different study) for validation in the AA cohort. Only miRNA 223-3p and miR-126-5p were well expressed across all samples. miR-19b and -106a were not sufficiently detected across patient samples from AA cohort and hence were not included in the analysis. Out of the profiled miRNAs, miR-223-3p, followed by miR-126-5p, showed the highest stability, as shown by the Reffinder rankings in Table 4. The combination of miR-223-3p and 126-5p was a better normalizer than individual miRNAs as shown by the stability rankings in Supplementary Table S1.

**TABLE 4:** Stability ranking of miRNAs in the validation African American cohort, single-tube qPCR data.

Method/Rank	1	2	3	4	5	6	7	8	9	10	11	12	13
Delta CT	miR-126-5p	miR-199a-3p	miR-223	miR-423-5p	miR-16	miR-126	let-7g	miR-29a	miR-30d	miR-885-5p	miR-376c	miR-26b	miR-142-3p
BestKeeper	miR-423-5p	miR-223	miR-126	let-7g	miR-126-5p	miR-199a-3p	miR-16	miR-29a	miR-885-5p	miR-30d	miR-376c	miR-26b	miR-142-3p
NormFinder	miR-126-5p	miR-199a-3p	miR-223	miR-423-5p	miR-16	miR-126	let-7g	miR-29a	miR-30d	miR-885-5p	miR-376c	miR-26b	miR-142-3p
geNorm	miR-199a-3p | miR-223		miR-423-5p	miR-126-5p	miR-16	miR-126	let-7g	miR-29a	miR-885-5p	miR-30d	miR-376c	miR-26b	miR-142-3p
Comprehensive ranking	**miR-223**	**miR-126-5p**	miR-199a-3p	miR-423-5p	miR-126	miR-16	let-7g	miR-29a	miR-30d	miR-885-5p	miR-376c	miR-26b	miR-142-3p

Among the four miRNAs miR-19b, miR-223, miR-106a, and miR-126-5p moved forward for validation, only miR-223 and 126-5p were sufficiently expressed in single-tube qPCR-based study in African Americans. Note: The additional miRNAs listed here are those selected from a previous single-tube qPCR-based study available to us, with good expression levels but are not endogenous controls.

## Discussion

MicroRNAs play important regulatory roles in health and disease states and thereby in treatment responses. Current lack of consensus on the data normalization strategies or a strong housekeeping microRNA for microarray data and qPCR data analysis impairs the validation of circulating miRNA associations in HTN and their translation as clinical biomarkers. Due to the variability in miRNA isolation profiling and analysis steps, clinical studies may lead to the identification of biased profiles, of which only subsets of the miRNAs clinical signature could be validated by RT-qPCR. Here, we identified the combination of miR-223-4p and 126-5p as a potential endogenous miRNA normalizer that can be used across microarrays and single qPCR assay platforms, as well as across ancestry groups, to control the technical variability across samples in the quantification of circulating miRNAs in HTN. We also provided a comparison of different normalization techniques and how they can affect the relative miRNA expressions, and in turn effecting the measure of true biological variations.

Currently, there is not a universal method that can efficiently reduce the technical variability in miRNA RT-qPCR data. Different techniques such as spike-in normalization, gene normalization, and small nucleolar RNA normalization have been used, but all these can lead to non-replicable results. The gold standard normalization is based on mean-centric methods and is very useful in high-throughput miRNA profiling, but it is not an option for analysis of a few miRNAs. In such cases, the use of endogenous controls is the best option (Santamaria-Martos et al., 2019). Given the differences in miRNA profiles in different disease states and tissues, circulating endogenous control miRNAs should be identified for each disease. The lack of a comprehensive analysis of normalizers for miRNAs in HTN patients could compromise miRNA results. For this reason, we performed miRNA array screening including 81 miRNAs as potential endogenous controls.

In our study, the quantile normalization followed by the global mean of all analyzed miRNAs data (with imputation of missing values) was identified as the best normalization strategy. Though quantile normalization has been previously shown to perform better than single-gene endogenous control normalization (Mar et al., 2009) or global normalization methods (Fundel et al., 2008) in terms of reducing the variation in the data, quantile normalization does not retain the true magnitude of expression differences across samples (Fundel et al., 2008) and is known to obliterate true biologically driven signals and generate false signals in downstream analysis (Wang et al., 2011; Zhao et al., 2020). Hence it may not be appropriate in biomarker discovery studies where associations of miRNA expression with phenotypes of interest are tested.

While some studies use global mean of the expressed miRNAs (omitting the missing values) as described first by Mestdagh et al. (2009), we identified that this method worked well only for the miRNAs with very good expression profiles and performed comparatively worse than the global mean normalization of all the analyzed miRNAs (imputing the missing values). Since it is known that circulating miRNAs can occur in low concentrations or might even be totally absent from the circulation in some individuals, a large number of missing values can be expected (De Ronde et al., 2017). Furthermore, it is known that with decreasing concentrations of the target miRNAs, the chance of finding a so-called “non-detect” increases (De Ronde et al., 2017). The RT-qPCR data contain a systematic bias resulting in large variations in the Ct values of the low-abundant miRNA samples. Complete exclusion of the missing data leads to the loss of data points resulting in a loss of statistical power. While there are several methods for imputation (which is beyond the scope of this article), in this study, we compared the utility of global mean normalization with or without imputation of missing data with Ct = 40. The calculated mean omitting the missing values could bias toward a good mean expression for samples with low miRNA expression, since the missing values of a majority of low-expression miRNAs are ignored, and the mean is calculated only from those expressed in a particular Ct range. In our study, MCR_norm and endogenous control normalization performed similarly, but a previous study for miRNA normalizers in the brain, placenta, and serum observed that MCR_mean performed better than the other types of normalization strategies in terms of reducing the SDs across the titration samples, while also showing maximum separation between true biologically different sample types (Wylie et al., 2011). Based on our data, we recommend using global mean normalization after imputation, as it has successfully reduced technical/experimental variability in the data, without purported masking of biological variability.

We identified miRNAs-223-3p, 19b, 106a, 126-5p as potential endogenous controls in a microarray miRNA profiling experiment and validated miRNAs-223-3p and 126-5p in the RT-qPCR–based single miRNA assay in an African American cohort. While it is possible that miRNAs 19b and 106a are downregulated in AAs, we cannot rule out the possibility of the lack of efficiency of the single miRNA qPCR probes used in this study. MiR-223-3p has been previously used as endogenous control (Kroh et al., 2010) due to its stable expression in plasma samples (Benson and Skaar, 2013). A previous study from our lab (Solayman et al., 2016) aimed to identify an endogenous control plasma miRNA for HTN, tested the stability of a set of five previously known reference miRNAs using a single assay qPCR-based method. While miR-223-3p showed the least stability among the tested candidates, it is important to note that the study compared both hypertensive and non-hypertensive patients, and some previous studies showed that miR-223-3p could be dysregulated in HTN and cardiovascular disease (Zhang et al., 2018; Zhang et al., 2021). Though serum- and platelet-derived miR-223-3p were shown to be downregulated in HTN patients, their consistent expression in the plasma promoted their utility as a biomarker for diagnosis of HTN with high sensitivity (Zhang et al., 2018). So, it is important to acknowledge that these findings may be applicable only in uncomplicated hypertensive patients.

MiR-19b was shown to be a good reference miRNA in an evaluation of seven potential normalizers in studies focused on cardiovascular diseases (Mar et al., 2009). It belongs to the miR-17/92 cluster that comprises miR-19b-1 and miR-17, and was also identified among the top 10 stably expressed miRNAs in our study. The miR-106a belongs to the 106a/363 cluster that also encodes miR-19b-2. Mir-106a has been previously used as endogenous control in different diseases (Sanders et al., 2012; Ortega et al., 2014; Schwarzenbach et al., 2015). We identified miR-126-5p as one of the consistently expressed miRNAs. A previous study by Pagacz et al. (2020) tested miR-126 as part of the set of reliable endogenous miRNA normalizers in the serum, in a variety of diseases, but not specifically in HTN. We further identified that the combination of miRNAs 223-3p and 126-5p was a better endogenous control than single miRNAs. This result is in line with previous studies (Li et al., 2015; Inada et al., 2018) that identified sets of two or more reference miRNAs to be better endogenous controls than single miRNAs. As stated in the MIQE guidelines for fluorescence-based quantitative real-time PCR experiments (Bustin et al., 2010), normalization should be performed with multiple reference genes, unless the single reference gene is sufficiently validated.

Limitations: The present study has several limitations. Our discovery cohort had a small sample size that might not truly capture the variability in miRNA expression across the patient population. But this limitation is circumvented by validating the consistency of miRNA expression in a larger, independent validation cohort that involved patients of different ancestry from discovery cohort, thus confirming the stability of miR-223-3p and 126-5p as housekeeping miRNAs across hypertensive patients. Nonetheless, it is to be noted that the validation cohort included only 13 miRNAs (that are not necessarily endogenous controls) for testing. Future validation studies should test miR-223-3p and 126-5p expression in comparison to other housekeeping controls to confirm their consistency and stability as endogenous controls. Multiple freeze–thaw cycles and age of the samples could negatively affect the stability of RNAs. Also, circulating miRNAs are oftentimes enclosed in extracellular vesicles and are considered to be relatively stable in extreme temperatures and freeze–thaw cycles. Nonetheless, the samples were stored and processed in aliquots, thus limiting the number of free-thaw cycles. Future studies could test the stability of miR-223-3p and 126-5p under multiple freeze–thaw cycles and with long-term storage. Low starting sample quantity and the age of samples could have reduced the quality of the RNA and the number of quantifiable miRNAs across samples. Only patients between 17 and 65 years of age have been studied, and larger studies should be performed to determine the validity of these endogenous controls in patients with different age groups, though our analysis showed that in the tested population, these miRNAs were not associated with changes in age or gender. We only selected the top four miRNAs from the discovery to test in the validation cohort. We acknowledge that there may be other more stable miRNAs in the AA cohort that might be better normalizers but were not tested in this study.

## Conclusion

The present study evaluated a variety of normalization strategies and identified global mean normalization as the most appropriate approach for microarray data. The results of this study also identified that the combination of miR-223-3p and 126-5p could be used as endogenous control for normalization of single-tube RT-qPCR–based miRNA profiling in essential HTN.

## Data Availability

The original contributions presented in the study are included in the article/Supplementary Material, and further inquiries can be directed to the corresponding author.
