# Supplementary material for: Comparison of Data Normalization Strategies for Array-Based MicroRNA Profiling Experiments and Identification and Validation of Circulating MicroRNAs as Endogenous Controls in Hypertension
Source: Front Genet. 2022 Mar 31;13:836636. doi: 10.3389/fgene.2022.836636 (PMC9008777; doi:10.3389/fgene.2022.836636)
Supplement: Supplementary file 1 [file DataSheet1.docx]

**Supplementary Figure-1: Trend in expression of normalizers in each sample from microarray data**

While mcr_mean, global mean of unimputed data and seem more consistent across patients, global mean of imputed data and miR-223 showed similar and stronger differences in expression between patients.

**Supplementary Table-1: Stability ranking of miRNAs in the validation African American cohort, single-tube qPCR data**

| Method | 1 | 2 | 3 | 4 | 5 | 6 | 7 | 8 | 9 | 10 | 11 | 12 | 13 | 14 |
| --- | --- | --- | --- | --- | --- | --- | --- | --- | --- | --- | --- | --- | --- | --- |
| Delta CT | mean223_126-5p | miR-126-5p | miR-223 | miR-199a-3p | miR-423-5p | miR-16 | miR-126 | let-7g | miR-29a | miR-885-5p | miR-30d | miR-376c | miR-26b | miR-142-3p |
| BestKeeper | miR-423-5p | mean223_126-5p | miR-223 | miR-126 | let-7g | miR-126-000451-b | miR-199a-3p | miR-16 | miR-29a | miR-885-5p | miR-30d | miR-376c | miR-26b | miR-142-3p |
| Normfinder | mean223_126-5p | miR-126-5p | miR-199a-3p | miR-223 | miR-423-5p | miR-16 | miR-126 | let-7g | miR-29a | miR-30d | miR-885-5p | miR-376c | miR-26b | miR-142-3p |
| Genorm | miR-223 \| mean223_126-5p | | miR-199a-3p | miR-126-5p | miR-423-5p | miR-16 | miR-126 | let-7g | miR-29a | miR-885-5p | miR-30d | miR-376c | miR-26b | miR-142-3p |
| Recommended comprehensive ranking | mean223_126-5p | miR-223 | miR-126-5p | miR-423-5p | miR-199a-3p | miR-126 | miR-16 | let-7g | miR-29a | miR-885-5p | miR-30d | miR-376c | miR-26b | miR-142-3p |

Mean223_126-5p = Mean of endogenous controls miR-223 and miR-126-5p
